# “If we can just dream…” Māori talk about healthcare for bipolar disorder in New Zealand: A qualitative study privileging Indigenous voices on organisational transformation for health equity

**DOI:** 10.1002/hpm.3486

**Published:** 2022-04-23

**Authors:** Tracy Haitana, Suzanne Pitama, Donna Cormack, Mau Te Rangimarie Clark, Cameron Lacey

**Affiliations:** ^1^ Department of Māori Indigenous Health Innovation (MIHI) University of Otago Christchurch New Zealand; ^2^ University of Otago Christchurch New Zealand; ^3^ Department of Public Health Te Rōpū Rangahau Hauora a Eru Pōmare University of Otago Wellington New Zealand

**Keywords:** bipolar disorder, health equity, healthcare organisation, healthcare quality improvement, Indigenous peoples, institutional racism, Māori

## Abstract

**Objectives:**

This paper identifies barriers to equity and proposes changes to improve the organisation of healthcare in New Zealand for Māori with bipolar disorder (BD) and their families.

**Design:**

A qualitative Kaupapa Māori methodology was used. Twenty‐four semi‐structured interviews were completed with Māori with BD and members of their family. Structural and descriptive coding was used to organise and analyse the data, including an analytic frame that explored participants' critique of attributes of the organisation of healthcare and alignment with Māori health policy.

**Results:**

Transformation to the organisation of healthcare is needed to achieve health equity. Executive management must lead changes to organisational culture, deliver an equity partnership model with Māori, embed cultural safety and redesign the organisation of healthcare to improve wellbeing. Healthcare incentive structures must diversify, develop and retain a culturally competent health workforce. Information management and technology systems must guide continued whole system improvements.

**Conclusion:**

This paper provides recommendations that should be considered in planned reforms to the organisation of healthcare in New Zealand. The challenge remains whether resourcing for an equitable healthcare organisation will be implemented in partial fulfilment of promises of equity in policy.

## Highlights

- Indigenous health equity requires the organisation of healthcare to transform.
- Executive management must lead changes to organisational culture.
- Change to healthcare culture will then support organisational re‐design.
- Evaluation, technologies, and incentives will support continued equity gains.

## 1 INTRODUCTION

Global health inequities between Indigenous and non‐Indigenous peoples remain prevalent despite equity objectives.^1^, ^2^, ^3^ In New Zealand neither ostensible commitments to Treaty of Waitangi rights or policies have transformed systems that privilege the majority European population and maintain inequitable health outcomes for Māori (Indigenous peoples of New Zealand).^1^ Donabedian's work identified an inextricable link between the organisation of healthcare and the processes and outcomes of care, with efforts at quality improvement requiring a focus on organisational change.^4^, ^5^ To date, efforts at healthcare improvement have largely focussed on the process and outcomes of care, while the significant upstream impact of the organisation of healthcare remains under‐researched and under‐addressed.^5^, ^6^

Health inequities between Indigenous and non‐Indigenous peoples are multifactorial, and include systemic failures to address differential exposures to and impacts of the social determinants of health, and unequal access to and through quality healthcare services.^2^, ^3^ Cultural competence and safety through organisational, structural, and clinical tiers of health systems is required to remove barriers to access, improve the quality of care and achieve equitable health outcomes for Indigenous peoples.^7^, ^8^ Improving the cultural competence and safety of healthcare organisations requires increased Indigenous leadership, and continued monitoring to inform the leadership approach and organisational changes required to drive health equity gains.^5^, ^7^

Glickman and colleagues^5^ developed a framework of five interrelated organisational attributes they considered fundamental to improving the quality of healthcare. The organisational framework identified the need for: Executive Management; Organisational Culture; Organisational Design; Incentive Structures; and Information Management and Technology to operate in sync to coordinate healthcare quality improvement work. Although Glickman and colleagues^5^ highlight the need for continuous monitoring and revision of healthcare quality improvements, the expertise of Indigenous peoples has not been adequately incorporated into organisational change.^6^

He Korowai Oranga^9^ is the Māori Health Strategy commiting the New Zealand Health and Disability Sector (H&DS) to health equity partnerships with Māori to achieve healthy Māori futures in healthy environments. Whakamaua^10^ is the Māori Health Action Plan to implement He Korowai Oranga through proposed changes to the organisation of healthcare, which can be mapped to the five organisational attributes of Glickman and colleagues'^5^ framework. Whakamaua proposes changes to mental health and addiction services as well as the wider health sector.^10^ Māori stakeholders have endorsed the need for organisational changes to mental health and addiction services, including stronger leadership, and a whole of government wellbeing approach to integrate health and social services.^11^ Although recommendations by Māori stakeholders are reflected in equity action frameworks, Māori patient and whānau expertise has rarely been utilised to identify problems and propose solutions to improve the organisation of healthcare.^6^, ^12^

The purpose of this qualitative paper is to identify barriers to equity and propose changes to improve the organisation of healthcare in New Zealand, informed by the experiences of Māori with bipolar disorder (BD) and their whānau. The Glickman and colleagues^5^ framework will be integrated with Whakamaua^10^ as an analytic frame to give voice to participants' organisational critique.

## 2 METHODS

### 2.1 Research approach and paradigm

Kaupapa Māori Research (KMR) methodology informed this qualitative study, and the broader research project this paper was drawn from.^13^, ^14^ KMR is a methodological framework that privileges Māori knowledges in the design, implementation and outcomes of research.^14^, ^15^ Methods aligned with KMR principles, to achieve the study aim and identify in this paper how organisational features of the health system influenced equity in health outcomes for Māori with BD and their family.^14^

### 2.2 Context

The New Zealand health system, though planning reform, is structured hierarchically, overseen by a centralised Ministry of Health.^16^ Services include: primary care by doctors in general practice; community‐based services; outpatient and inpatient hospital services delivered regionally by 20 District Health Boards and non‐governmental organisations. Mental health care for BD generally requires a general practice referral to District Health Board services, and can include periods of inpatient or community‐based treatment by multi‐disciplinary teams within a psychiatric care model. Services and teams can differ between districts, meaning experiences of care may vary depending on where in New Zealand a person lives.^17^ The focus of inquiry for this paper will explore the whole spectrum of Māori patient and whānau experiences of receiving health services for BD, from primary healthcare to specialist hospital care.

### 2.3 Sample

Twenty‐four semi‐structured interviews were completed across three New Zealand sites selected for their range of mental health services, rural and urban loci. Table 1 summarises self‐reported demographic information for Māori patients with BD (*n* = 24) who participated. Over half of interviews included the perspectives of patients together with one or more members of their whānau (family/support network; *n* = 19). All patients had a BD diagnosis with stable mood at interview. Mental health staff gave study information to eligible patients, and interested participants were recruited by the research team. No exclusions were made for co‐morbidities. A purposive sampling frame recruited men and women of differing ages across sites. Participants provided written informed consent before interviews.

**TABLE 1:** Participant demographics for Māori patients with bipolar disorder

	Interviews (*n* = 24)	Percentages
**BD diagnosis**		
Type I	20	83.3%
Type II	2	8.3%
NOS	2	8.3%
**Inpatient admissions**		
Yes	22	91.7%
No	2	8.3%
**Whānau at interview (n = 19)**		
Yes	13	54.2%
No	11	45.8%
**Psychiatric comorbidity**		
Yes	12	50.0%
No	12	50.0%
**Physical comorbidity**		
Yes	9	37.5%
No	15	62.5%
**Age range**		
16–24	2	8.3%
25–44	8	33.3%
45–64	12	50.0%
65+	2	8.3%
**Gender**		
Men	10	41.6%
Women	14	58.3%

Abbreviation: NOS, not otherwise specified.

### 2.4 Ethics

Ethical approval was received from the Health and Disability Ethics Committee of New Zealand (ID:16/STH/137). The CONSolIDated critERia for strengthening research involving Indigenous peoples (CONSIDER statement) aligned the study with Indigenous research guidelines and priorities.^13^, ^18^

### 2.5 Procedure

Interviews were conducted in‐person by two of the research team between December 2017 and August 2019 at participants' homes, health services, or a research unit. The interview schedule was informed by a systematic literature review,^19^ and adapted cultural competence framework.^20^ Questions explored the impact of organisational features of the health system on participants' wellbeing.

### 2.6 Data collection and processing

Interviews were recorded, transcribed and analysed by authors. Transcripts were anonymised assigning numbers to each interview (1–24), participant (P1‐P24) and their whānau (W1‐W24). Where multiple whānau were present, an interview number and letter was assigned (W1a, W1b, W1c). NVivo12 software was used to display transcripts, code data, and refine codes, categories and themes and monitor saturation across themes and sub‐themes.

### 2.7 Data analysis

Two coding cycles were completed.^21^ Cycle one involved two phases. Phase one used a structural coding method and an adapted cultural competence framework from Betancourt and colleagues^20^ to group data into participants' critique of clinical, structural, and/or organisational features of the New Zealand health system.

Betancourt and colleagues^20^ defined the ‘organisational’ component of the health system to include the *leadership that design health care systems and processes and the workforce that implement them*. During phase one coding, the criteria for inclusion widened to incorporate *theory, policy and leadership that informs health system priorities*, *design and delivery of care to Māori patients and whānau, including how health and other social systems align, and who is included in the health workforce.* For this paper, only findings and analysis from the grouped ‘organisational’ code drawn from Betancourt and colleagues'^20^ analytic frame will be presented.

Phase two also used structural coding, and re‐grouped the organisational data with an adapted coding framework integrating Whakamaua^10^ with Glickman and colleagues'^5^ five organisational attributes for healthcare quality improvement. Supplementary Table 1 summarises the adapted coding framework employed in phase two. Cycle two used descriptive coding to identify topics within the data that were grouped into the five organisational codes.

Coding notes were recorded during phase one and two to synthesise the detail contained within coded transcripts. Notes were displayed within a table, reviewed by authors, and reorganised according to whether they reflected problems within the existing organisation of healthcare or proposed solutions to these. Coding notes were then arranged into the descriptive codes for the five organisational attributes of healthcare quality improvement.

### 2.8 Data display

A summary of descriptive codes evident within the organisational principles of Executive Management, Organisational Culture, Organisational Design, Incentive Structures and Information Management and Technology is provided. For each descriptive code a summary of participants' critique of problems in the organisation of healthcare is reported, and quotes used to illustrate these. At the end of the problem summary, synthesised coding notes are presented in tables for each descriptive code proposing organisational changes to improve healthcare for Māori with BD. Tables represent a synthesis of participants' critique taken directly from transcripts and the process of analysis, with some proposed organisational solutions inferred from identified barriers. Language within the tables is framed to reflect the prestige of participants and presents suggestions for a strengths‐based organisational redesign for healthcare.

## 3 RESULTS

**FIGURE 1:**
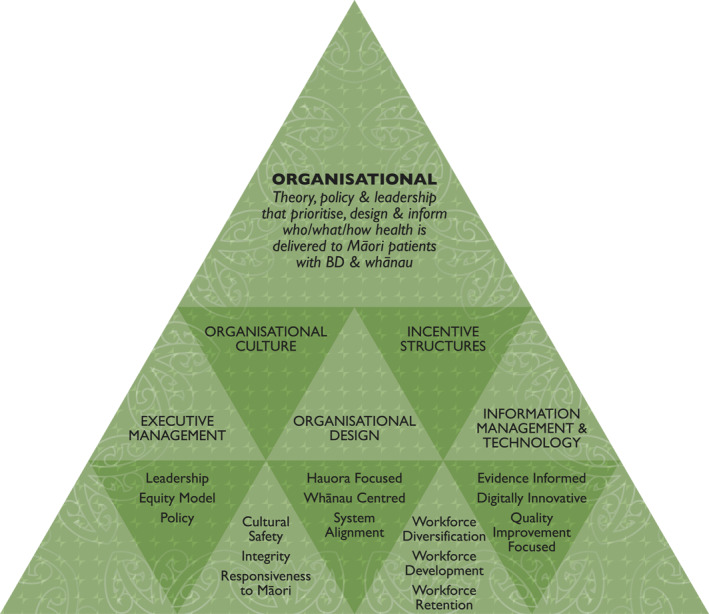
Codes and sub‐themes from critique of organisational features of the health system

Participants' critique aligned with organisational codes exploring the extent to which features of the current New Zealand Mental Health System (MHS) reflected Whakamaua from the perspectives of Māori receiving BD services. Three sub‐themes from each of the five organisational codes were identified from the analysis. Figure 1 illustrates organisational codes and their related sub‐themes.

### 3.1 Executive management

Three sub‐themes were identified from the Executive Management code, including participants' critique about: Leadership; the need for an Equity Model; and the implications of Policy on healthcare.

#### 3.1.1 Leadership

Participants considered that the MHS needed to ensure the executive management and leadership teams were culturally competent to establish effective partnerships with iwi (tribal groups), and achieve equity objectives for Māori with BD.

Māori have the capability, but the system needs the capacity and strength to have courageous conversations about our people to work with Iwi to enhance their own hauora (wellbeing). (P1)

Participants reported that the executive management team needed to proactively support and increase the capacity of Māori to move into leadership positions to improve the responsiveness of the MHS for Māori with BD and their whānau.

We want the best person for the job but there's no reason why Māori can't be upskilled to be the best person for the job. They may not start out in an equal space, but we need to put things in place to support them. Māori deserve the best and it should be by Māori for Māori. (W1 and P1)

Participants considered the executive management team must acknowledge the need for whole system change, then partner with Iwi and other health‐related sectors, adapt models of care and redistribute resources to ensure the system did not continue to underserve Māori.

The current model needs to be influenced by the data that shows Māori are all in the negative statistics, with an honest attempt and proper resourcing and funding to work for Māori. (P2)

Participants' critique of organisational leadership revealed opportunities for change. Attributes of effective leadership in a healthcare organisation redesigned to achieve equitable health outcomes for Māori with BD and their whānau are presented in Table 2.

**TABLE 2:** Attributes of organisational leadership for healthcare equity

• Acknowledge the ongoing impacts of colonisation on wellbeing for Māori and explicitly commit to equity in H&DS outcomes.
• Establish co‐governance partnerships with iwi and collaborate with Māori leadership groups in the healthcare organisation and other health sectors.
• Normalise partnerships with Māori throughout the H&DS with more Māori in leadership roles modelling expected clinical partnerships with patients/whānau.
• Establish Māori leadership equity pathways resourced to increase Māori leadership capacity.
• Oversee the collection of quality Māori health data and review progress towards health equity with iwi partners to refine continuous organisational healthcare improvements for equity.

#### 3.1.2 Equity model

Participants recognised that Western Science, through the psychiatric model, dominated the organisation of the MHS influencing the processes, delivery and focus of services in ways that did not support health equity for Māori with BD. The psychiatric model was criticised by participants, because of the narrow focus on sickness, restricting service access, and prioritising medications and risk management without integrating Māori models of health.

Māori were my absolute authority. They contrast the way the Pākehā (Mainstream/Western/European) system has treated me. The hierarchy, with psychiatry at the top has to flatten out. You see tangata whaiora (Indigenous people seeking wellness) now, so medicated, their wairua (spiritual/non‐physical dimension of wellness) taken off them. (P3)

Participants identified that an equitable MHS required executive management to address biases and remedy systematic failures in the current organisation of health care. An equity model therefore required coordinated and targeted resourcing aligning the H&DS, minimising the impact of greater exposures and vulnerabilities to health compromising conditions impacting Māori with BD and their whānau.

We wanted her to have somebody outside of us to trust, to express herself with, and to look at the injuries she had suffered. They just labelled her ‘bipolar’ and didn't want to know the rest. (W2)

Participants' critique of the dominant Western model of the organisation of healthcare revealed opportunities for change. Attributes of an organisational model redesigned to achieve equitable health outcomes for Māori with BD and their whānau are presented in Table 3.

**TABLE 3:** Attributes of an equity model of healthcare organisation

• Integrate Māori knowledges with Western Science enhancing wellbeing for Māori patients with BD and their whānau.
• Oversee funding adjustments to reprioritise services for Māori to achieve health equity.
• Oversee an extension from diagnostic to Māori models of healthcare, coordinating whānau‐centred holistic service provision including care outside/parallel to the H&DS.
• Fund and resource expansions to early intervention and relapse prevention within the organisational design of the MHS.
• Implement provision for healthy traditional Māori food sources, practices to restore a person's wairua, tohunga (Māori health practitioners), family wellbeing, and social welfare needs by aligning a whānau‐centred, whole health sector care model.

#### 3.1.3 Policy

To achieve health equity for Māori with BD, it was evident from the experiences of participants that existing MHS policies required substantial revision. Revisions needed to integrate Māori knowledges in all legislation, policies and procedures across the MHS, including the Mental Health Act, to facilitate working in partnerships to maintain the autonomy of Māori patients and whānau.

It's not nice, the way they come at you, it needs to be way different. If it was different you wouldn't be so angry at the system, if you knew what it was that they were going to do and they did it with you. (P4)

Participants noted that services designed to improve outcomes for Māori needed to have the capacity and resources to work with Māori patients with BD and whānau differently. An equity model therefore required changes to health policy to be implemented across the H&DS to ensure meaningful and tangible service adaptations to meet the needs of Māori patients with BD and their whānau.

I think if it was offered that we had Māori Health Services available that provide you with better care or more specialised care or something like that, whereas it currently comes across as just a brown interpretation of the same system. I don't need to tick the brown box. (P5)

Participants' critique of organisational policy revealed opportunities for change. Attributes of effective policy in a healthcare organisation redesigned to achieve equitable health outcomes for Māori with BD and their whānau are presented in Table 4.

**TABLE 4:** Attributes of organisational policy designed for healthcare equity

• Implement policies developed by Māori leadership in partnerships with iwi.
• Embed principles like Māori sovereignty and Māori models of health into legislation to guide patient/whānau privacy, and compulsory treatment and assessment orders under the mental health Act.
• Establish inter‐agency, cross‐service policy agreements (agencies mentioned included ACC [Accident compensation corporation, a no‐fault accidental injury compensation scheme], Oranga Tamariki [child care and protection agency], social welfare, Police, and other non‐government services) to uphold the respect and dignity of patients/whānau through shared care, harm‐prevention plans.
• Upgrade information‐management and technology to support hauora‐focussed policy and facilitate timely information sharing and multi‐agency/service care planning.

### 3.2 Organisational culture

Three sub‐themes were identified within the Organisational Culture coding, including participants' critique about: Cultural Safety; Integrity; and Responsiveness to Māori.

#### 3.2.1 Cultural safety

Participants reported that culturally safe, competent care was not integrated into the organisational culture of the MHS. The lack of integration was described by Māori patients and whānau as an ‘add on’ to ‘standard’ clinical approaches. Clinical approaches dominated participants' experiences of BD care, with cultural safety described as an exception rather than a normative feature of the H&DS.

I was privileged initially to see the Whare Tapa Whā model (a holistic Māori model of health). I felt comfortable through the use of whakawhanaungatanga (process of reciprocal relationship building), my parents understood and had an explanation from a Māori doctor. Since then though, how far have we actually come? (P2)

The availability of Māori staff was viewed by participants as pivotal to the cultural safety of the MHS, through their understanding and expression of Māori knowledges. Participants felt that Māori staff were under‐resourced in the MHS. To support cultural safety improvements and normalise cultural safety across the H&DS, a larger Māori workforce was needed, in more diverse roles with responsibilities tailored to achieve health equity for Māori.

If they want to make a difference for Māori with bipolar, health systems need to go deeper. They need to value the spirituality of our cultural being. Māori providers involved with inter‐sectoral engagement is needed to support the expansion of health and social sectors. (W3)

Participants' critique of cultural safety in healthcare revealed opportunities for organisational change. Attributes of a culturally safe healthcare organisation redesigned to achieve equitable health outcomes for Māori with BD and their whānau are presented in Table 5.

**TABLE 5:** Attributes of cultural safety in an equitable healthcare organisation

• Integrate Māori knowledges with clinical knowledge to guide revised processes and standards of care for patients/whānau across the whole H&DS.
• Allocate resources across the H&DS to normalise culturally safe and competent care by all services/regions and staff in all roles.
• Increase capacity and diversify roles of Māori staff using incentive structures that recognise and reward Māori knowledge competencies.

#### 3.2.2 Integrity

Participants reported that the organisational culture required restorative processes to rebuild trust and repair partnerships with Māori patients and whānau following harmful contact with the MHS. Honesty and accountability were considered by participants as essential in the culture of the MHS, especially for relapsing conditions like BD, to remove barriers from re‐engaging when required.

I don't really go there anymore. I try to keep out of the hospital. I can't trust people. My psychiatrist is helpful, she counsels me. She's real I feel. Some people aren't. (P6)

Due to the dominance of the psychiatric model, enhancing wellbeing and preventing BD relapses were not prioritised in the organisational culture of the MHS. Participants expressed frustration about the illness focus of the organisational culture, and the failure of the MHS to acknowledge or address the impacts of the social determinants of health on Māori patients with BD and their whānau. The illness focus perpetuated mistrust and maintained power imbalances between the MHS, Māori patients and whānau. Participants' experiences reflected a lack of integrity in establishing meaningful partnerships with Māori patients and their whānau in the current organisational culture of BD services.

I'm determined not to go back to hospital. It's not just a genetic thing. Doctors dismiss the patterns. It's just get in, get your medication, heal yourself, get out. (Staff) never address the reasons why. (P7)

Participants' critique of the integrity of the organisational culture of healthcare revealed opportunities for change. Attributes of integrity in a healthcare organisation redesigned to achieve equitable health outcomes for Māori with BD and their whānau are presented in Table 6.

**TABLE 6:** Attributes of integrity in an organisational culture designed for health equity

• Evaluate the H&DS and be accountable to Māori patients with BD/whānau to increase trust and build effective partnerships between services, clinicians and Māori communities.
• Acknowledge the limitations of the psychiatric model for Māori by reorienting to an equity model of care with an expanded scope of hauora‐focussed services.
• Address the impact of the social determinants of health by pooling resources in partnerships between Māori patients/whānau and other services/agencies.

#### 3.2.3 Responsiveness to Māori

Participants reported that the organisational culture of the MHS was often not responsive to the needs of Māori patients with BD or whānau. The organisational culture was criticised for failing to adequately resource, support and review staff training needs to ensure a culturally safe and competent workforce. Participants' encounters with services reflected the dominance of the psychiatric model and lack of integration of Māori knowledges into the organisational culture. Effective partnerships between the MHS, Māori patients with BD and whānau were therefore limited by policies and procedures that essentially excluded meaningful integration of Māori knowledges into the existing model of service provision.

New medication gave me a bit more life so I felt awake, and then I started learning te reo (Māori language) which opened up another pathway to mau rākau (Māori martial art) together with my whānau. Cutting that mainstream stuff out and bringing in structure from te ao Māori (the Māori world) helps me stay well. (P8)

The organisational culture was considered by participants to focus on illness and risk for individuals, without understanding or introducing supports of relevance to the wider whānau.

There was no real support for us as whānau as to how we could best support him, what was best to do, or what his condition was. He had been unwell for nearly a year, in treatment for eight months by the time we actually got family support. (W4)

In addition, the organisational culture was not resourced to support cross‐team or cross‐service collaboration, leading to over‐specialisation of services to the detriment of Māori patient and whānau wellbeing.

There was conflicting advice, arguments about which condition was worse. My Māori health worker was the only consistent person. Services all had separate priorities. They can't be separate with one person in between. (P9)

Participants' critique of the responsiveness of the organisational culture to Māori revealed opportunities for change. Attributes of an organisational culture redesigned to improve responsiveness to Māori with BD and their whānau are presented in Table 7.

**TABLE 7:** Attributes of an organisational culture redesigned to be responsive to Māori

• Resource, support, review, and normalise ongoing cultural safety and competency training for staff across the H&DS with skills to respond to diverse Māori realities, faith practices, and to recognise and address racism in healthcare.
• Value mātauranga Māori alongside Western Science evidenced by partnerships with Māori and non‐Māori staff, patients with BD and whānau, and cross‐team/cross‐service/cross‐agency/community relationships for whānau‐centred healthcare.
• Embed Māori models of health integrating mental and physical healthcare, early intervention/relapse prevention, and partnerships with services/patients/whānau.
• Establish cross‐service specialist teams/leadership to educate/resource staff to implement shared care plans for Māori patients/whānau with comorbid conditions (e.g. substance use, eating disorder, trauma‐informed care, physical health care, etc).

### 3.3 Organisational design

Three sub‐themes were identified from the Organisational Design coding, which included participants' critique about the extent to which mental health services were: Hauora‐Focussed (Wellbeing‐focussed); Whānau‐Centred; and facilitated System‐Alignment.

#### 3.3.1 Hauora‐focussed

Participants critiqued the current organisational design and reported that the MHS perpetuated stigma and stifled wellbeing for Māori with BD through an over focus on illness. The organisational design of the MHS was experienced by many participants as aversive, to be avoided where possible because the organisation was deemed to prioritise ‘risk’ and ‘medication adherence’ more than wellbeing.

If you are only talking about depression and anxieties and ailments, damn straight you are going to bring them into the next day. You won't be able to get out of bed, because you are not talking about strategies and how you got out of bed this morning. The focus is deficit, not how to rise. (P10)

Some participants raised the need for the MHS to be tailored to identify and address the diverse realities of Māori whānau, including those who may not be socially ascribed as Māori. Participants noted that understanding the diverse realities of Māori required a more flexible, integrated organisational design. Services integrating an equity model of care were preferred by some Māori participants to prevent feeling marginalised by having to choose between ‘mainstream’ or Kaupapa Māori services. In particular, a more integrated organisational design was preferred by participants whose access to Māori knowledges was affected by the impacts of colonisation.

That affects me when it comes to choosing services, who do I want? I can't identify strongly with either. They need to understand that we're made up of different cultures. We're not just one or the other and we might not identify strongly with any culture. And that's both Māori and Pākehā. (P11)

The current organisational design was also reported by participants to perpetuate repeated loss of relationships due to multiple care transitions between staff and services. Minimising transition points, or providing continuity of care, was valued by participants, although it was not a consistent feature of the organisational design.

You can't wait for weeks for services to go through their processes. You need help when you need it. We have support now through Māori staff outside of those services, because trusting people is an important issue. Trusting who is going to work with him, and who he gels with. (W5)

Participants' critique of problems in the organisational design of healthcare also revealed opportunities for change. Attributes of a hauora‐focussed organisation redesigned to achieve equitable health outcomes for Māori with BD and their whānau are presented in Table 8.

**TABLE 8:** Attributes of a hauora‐focussed organisational design for healthcare equity

• Resourced to extend the H&DS outside of hospitals and into communities, by introducing traditional Māori food growing and gathering practices co‐run by/with/for Māori patients/whānau.
• Partner with social enterprises to enhance knowledge/skills/autonomy of Māori patients with BD and their whānau extending into employment opportunities building on Māori food growing and gathering practices to supply healthy food for hospitals and communities in need.
• Align with changes to the organisational culture and incentive structures by resourcing and training the H&DS to identify and address the impacts of New Zealand history, racism and marginalisation on wellbeing for patients/whānau.
• Prioritise partnerships between Māori patients/whānau/staff/services, by monitoring their performance using information management and technology, and recognising/reinforcing culturally safe/competent staff/services and incentive structures.

#### 3.3.2 Whānau‐centred

Participants reported that the current organisational design of the MHS relied on whānau to support Māori with BD to access services, but after admission, legislation and the patient‐centred model of care marginalised and excluded whānau input.

When he got admitted it was horrible. I knew Dad would hate me for it, but it had to be done. They were fine with me admitting him, but they weren't helpful at all. I was left with that responsibility. (W6)

Participants emphasised that the MHS was fundamentally designed to be delivered to individual patients, even if clinicians and services sought to include Māori knowledge elements. This meant that services were not whānau‐centred by design, and lacked the resources to support whānau who were essential to the wellbeing of Māori with BD.

When Mum ended up in the unit it was scary, sad, confusing. We didn't understand. Services didn't support us in any way. It was worrying and we didn't have anyone we could talk to. It was just something that was happening to our Mum. (W7)

Participants' critique of problems in the organisational design of healthcare revealed opportunities for change. Attributes of a whānau‐centred healthcare organisation, redesigned to achieve equitable outcomes for Māori with BD and their whānau are presented in Table 9.

**TABLE 9:** Attributes of a whānau‐centred organisational design for healthcare equity

• Deliver healthcare across the H&DS aligned with Māori models of health.
• Deliver whānau‐centred interventions to increase wellbeing using processes like whakawhanaungatanga to provide information about BD, and build partnerships with services that empower Māori patients/whānau.
• Develop new roles and responsibilities for existing MHS staff, and increase cross‐service/agency collaboration to resource Māori patients without whānau supports.
• Adopt an equity model with greater resources for cross‐team/cross‐service and whānau‐centred care plans across the H&DS.
• Integrate Māori models of health that prioritise family wellbeing across the whole H&DS in both ‘mainstream’ and kaupapa Māori services.

#### 3.3.3 System‐alignment

Participants expressed frustration about the separation of mainstream health and social systems and considered that this design perpetuated inequities for Māori with BD, forcing them to navigate multiple services, often during the same period of illness.

If we can just dream, it would be that people with mental illnesses who are unwell could be offered the services they need. So, for example, I am on an unemployment benefit with a medical certificate. But I have to ask my GP (General Practitioner) every month for a medical certificate. It would be nice if that was somebody else's job. (P5)

Misalignment in the organisational design of health systems was considered an inefficient use of limited resources by participants, reducing hope and trust in the MHS, and contributing to staff burnout across the H&DS. Systemic misalignment had a particularly adverse impact on young people with BD, as the lack of coordinated support and resources compounded stress at home, in educational settings, in whānau and within peer groups.

We found out he stopped medication when he got the sickest. His flatmate rang and that is when he dropped out of University. We were navigating the system blindly. We didn't know what the GP could do versus what the mental health team could do or how to access mental health. (W4)

Participants' critique of problems in the organisational design of healthcare revealed opportunities for change. Attributes of effective system‐alignment in a healthcare organisation redesigned to achieve equitable health outcomes for Māori with BD and their whānau are presented in Table 10.

**TABLE 10:** Attributes of organisational design using system‐alignment for health equity

• Deliver whānau‐centred, hauora‐focussed healthcare by operating across services/systems/agencies and generations of whānau.
• Pool limited resources across the whole system to facilitate access to and through timely/quality healthcare for Māori patients with BD and their whānau.
• Implement health and justice policy reforms that prevent the criminalisation and aversive inpatient admissions of Māori patients with BD.
• Implement wellbeing programmes to support Māori youth with emerging mental health difficulties, including BD, and enhance family wellbeing.

### 3.4 Incentive structures

Three sub‐themes were identified from the Incentive Structures code, including participants' critique about: Workforce Diversification; Workforce Development; and Workforce Retention.

#### 3.4.1 Workforce diversification

Participants highlighted that their experience in the MHS would be enhanced by increasing the cultural competence of the workforce. Participants also considered that the MHS needed to increase the Māori workforce and diversify their roles to improve wellbeing for Māori patients with BD and whānau.

(Staff) need to be approachable, not clinical. I want to see Māori staff before and after what's about to take place. Explain what they're going to ask and say. Acknowledge you're not feeling well, and if you want to stop, have a cup of tea and something to eat, and debrief afterwards. You need barriers broken down and time for (staff) to be real people. (P12)

Participants considered medical professionals were overvalued due to the dominance of the psychiatric model, and advocated instead for a workforce with training, knowledge and expertise in Māori health models of best practice for BD.

No point increasing the workforce just because they're Māori, but bringing out their innate ability to engage with Māori clientele. We talk about the Whare Tapa Whā model and wairua, but do we actually manifest that in our services? We need to have nationwide providers, an ongoing wellness plan, and the best Matakite (Māori healers) to be part of that. (P1)

Participants' critique of problems with incentive structures in the organisation of healthcare revealed opportunities for change to increase workforce diversification. Attributes of a healthcare organisation with incentive structures designed to build a diversified workforce to provide equitable care to Māori with BD and their whānau are presented in Table 11.

**TABLE 11:** Organisational incentive structures to diversify the healthcare workforce for equity

• Prioritise applicants with cultural safety/competency/Māori knowledge skills during recruitment of staff for the H&DS.
• Establish training pathways for graduates and overseas trained staff to equip those entering the health workforce with cultural safety/competency knowledge/skills to deliver best practice for BD with Māori patients/whānau.
• Establish equity pathways into training programmes that prioritise health workforce recruitment increasing Māori representation in the H&DS.
• Diversify roles/responsibilities for Māori in health supporting the delivery of Māori models and approaches to health (e.g. knowledges of Māori customs, protocols and practices, Māori methods of healing, and Māori language expertise).
• Increase the capacity of services to deliver best practice treatments for Māori with BD, including greater numbers of staff to provide psychological interventions.
• Establish new roles for Māori health navigators and non‐clinical staff to orient Māori patients/whānau to BD services/processes and facilitate cross‐service/system care plans.

#### 3.4.2 Workforce development

Participants noted that their experiences were not utilised by the MHS to address knowledge, training and competency gaps or biases within the health workforce. Participants identified that the MHS needed to invest in the development of Māori and non‐Māori staff to improve the capacity of the mental health workforce to deliver safe, effective and equitable BD care in partnerships with Māori.

I'm going to be blunt. There needs to be relevant education. If people haven't got the heart to deal with our afflictions and they are not passionate about it, don't be in the job. Simple. (P10)

Some participants expressed frustration about variations in the standards of care they received across the MHS, differing between regions and teams.

I don't know if it is just different regions or changes over time, but after four visits, my psychiatrist is trying to discharge me. I have no social work support, when I previously had help every week sourcing things I needed in my area. Here I have had none of this. (P5)

Participants considered that greater monitoring and resourcing was required to ensure Māori patients and whānau received consistent care from a culturally competent mental health workforce, in line with human rights, in all regions, services and teams.

In the ward, the night shift were cruel. I saw how they treated the patients in there. I got thrown in the lock up. I'd wait until the day shift. They were always smiling. Most of those staff are not here now. (P13)

Participants' critique of problems with incentive structures in the organisation of healthcare revealed opportunities for change to improve workforce development. Attributes of a healthcare organisation with incentive structures designed to develop a competent workforce delivering equitable care to Māori with BD and their whānau are presented in Table 12.

**TABLE 12:** Organisational incentive structures to develop the healthcare workforce for equity

• Embed a growth mindset into the organisational culture of the H&DS reinforced by incentive structures that normalise development and reflective practice.
• Measure baseline skills/knowledge/capacity of the health workforce to guide staff development supporting healthcare quality improvement/equity gains for Māori with BD and their whānau.
• Resource ongoing training/evaluation supporting the development of a culturally competent and safe health workforce.
• Gather feedback from Māori patients/whānau using information management and technology to improve responsiveness/partnerships with all teams/services.
• Regularly monitor efficacy of staff/teams/services during Māori patient/whānau contact with the H&DS to refine healthcare quality improvements and workforce development.
• Complete restorative justice processes after harmful service contacts to guide workforce development and achieve health equity for Māori.

#### 3.4.3 Workforce retention

Participants identified the importance of incentive structures to retain staff effective in working with Māori patients with BD, who made them and their whānau feel welcome and valued within the clinical setting.

I've had contact with Māori services. They were really good, the people that were there. It was like being in a normal situation, not a hospital or clinical setting. It was familiar and comfortable. It doesn't really matter whether you are in a Māori or mainstream service if there are good people in both. (P14)

Receiving care from a Māori clinician was seen as desirable by participants, however they noted that mainstream policies and procedures restricted the ability for both parties to engage in cultural processes in a fluid and natural way to facilitate cultural safety.

On the ward, some staff talked to you and treated you as one of them. But in Kaupapa Māori teams, the whakawhanaungatanga was good. We'd call them uncle or aunty. Some worked for mental health for 15 years, they're gone now, but everyone still knows them. (P13)

Participants identified that limitations of organisational policies and procedures often led to Māori and non‐Māori staff who were culturally competent, being marginalised by their team and created frustration about the way services were delivered. The outcome often meant culturally competent staff were not retained in services.

I have seen a lot of really good staff who aren't supported by their co‐workers or the organisation – they don't get the credit for the work that they do. The system can be quite brutal to staff. (P14)

Participants' critique of problems with incentive structures in the organisation of healthcare and the impact on workforce retention revealed opportunities for change. Attributes of a healthcare organisation redesigned to improve incentive structures, and retain a competent workforce to provide equitable care to Māori with BD and their whānau are presented in Table 13.

**TABLE 13:** Organisational incentive structures to retain a culturally safe healthcare workforce

• Utilise incentive structures to recognise and reward staff who work effectively with Māori patients with BD and their whānau in the H&DS.
• Ensure executive management, organisational culture and design level changes occur to retain a culturally safe/competent workforce and prevent staff burnout.
• Implement processes and procedures that support Māori staff, patients with BD and whānau to manage dual relationships/genealogical/community connections.
• Introduce flexible contracts/working conditions to retain culturally competent staff.
• Introduce workforce development, supervision and supports to enhance staff wellbeing, retain and continuously grow a culturally responsive workforce.
• Diversify roles/responsibilities aligned with equity objectives by offering training pathways to support Māori staff to implement Māori models of healthcare.

### 3.5 Information management & technology

Three sub‐themes were identified from the Information Management and Technology coding, which included participants' critique about the extent to which the healthcare organisation prioritised the use of information management and technology to be: Evidence‐Informed; Digitally Innovative; and Quality Improvement‐Focussed.

#### 3.5.1 Evidence‐informed

Participants expressed frustration that the MHS did not draw on evidence from Māori patient or whānau experiences, or recognise their value in informing organisational changes, particularly after harmful contacts with services or staff.

Mental health services treated her as a threat. That reputation was on file and they would ring security for no reason. They need to take time to really listen, because you get brushed off. They say “oh yeah, yeah” but nothing ever changes. (W8 and W9)

Participants' experiences illustrated that the organisation of healthcare privileged evidence from Western Science through the hierarchy of the psychiatric model, negating the expertise of Māori with BD and their whānau.

I wanted access to a psychologist because I didn't think I needed anti‐depressants. I asked for years but never got it. I'm sure the nurses inadvertently support the lack of movement because they can't change anything when they see you. So they ask how your pills are going, but just report back to the psychiatrist that they are fine. (P3)

Through this critique, participants saw gaps between the current organisational structure and an equity model where Māori knowledges are valued equally, informing evidence‐based care and service innovations. Even when participants invested substantial time and energy to give feedback, without the oversight and support of Māori leadership they found no commitment to organisational change.

I contacted the Human Rights Commission. I made a complaint. I got a letter back saying it was wrong, but it all came back to me to do something about it. They said ‘yes it's wrong’, and sent it back to me. There has been no apology. Nothing. (P3)

Participants critiqued the influence of information management and technology on evidence informing the organisation of healthcare revealing opportunities for change. Attributes of an organisation redesigned to use information management, and technology to evidence equitable healthcare for Māori with BD and their whānau are presented in Table 14.

**TABLE 14:** Attributes of an evidence‐informed organisation for healthcare equity

• Invest in information management and technology strategies so leadership partners receive feedback from Māori patients/whānau to inform healthcare quality improvements across the organisation.
• Use information and technology to monitor, evaluate and improve the quality of care for Māori with BD by comparing services against best practice standards.
• Monitor and evaluate the quality/efficacy of holistic cross‐service care for Māori engaged with multiple teams to support healthcare quality improvements across the organisation of healthcare.
• Use KMR experts to develop/monitor/analyse and relay Māori service use data to leadership teams to develop the organisation of healthcare to achieve equity.

#### 3.5.2 Digitally‐innovative

Some participants utilised digital health technologies resourcefully, and recognised ways that information management and technology could be harnessed to improve the management of chronic conditions like BD. However, participants expressed frustration when data they gathered was disregarded by services in favour of clinician knowledge, or subjugated by fixed organisational processes. Participants' experiences highlighted ways that better use of information management and technology could improve responsiveness to Māori with BD, and progress towards health equity.

I know how I work, why my brain does that, how I respond to medication. I use e‐moods and my Fitbit to monitor everything. I contacted services and said I slept 15 hours yesterday, I went from eight to 15 hours in two weeks, the medications are over‐sedating me now. Please tell the psychiatrist. They said the psychiatrist advised to keep taking the same amount. (P5)

Participants also expressed frustration about delays and inefficiencies in their assessment or BD treatment due to a lack of digital‐innovation in the storage, management and sharing of patient health information.

No one would listen to me. They wouldn't take me further than the desk. I was diagnosed ten years ago. I hadn't used New Zealand services until I came back to the country last year. I had to act like a maniac just to get someone to take me seriously. (P15)

Through the critique of participants, it was also clear that the MHS could better utilise digital‐innovations to monitor adherence by services to best practice standards for BD treatment for Māori.

I'm on sleeping pills and another drug that was only supposed to be given to me for a month after I'd come out of hospital. I've been on them for nearly two years and now they're only just trying to wean me off. That's ridiculous. (P16)

Participants' critique of digital‐innovations used to inform the organisation of healthcare revealed opportunities for change. Attributes of an organisation redesigned to use digitally innovative information management and technology to achieve equitable healthcare for Māori with BD and their whānau are presented in Table 15.

**TABLE 15:** Attributes of a digitally innovative organisation designed for healthcare equity

• Equip Māori patients with BD and their whānau with digital health aids, to track symptom changes, strengthen partnerships with the H&DS and proactively manage mood states.
• Strengthen information‐sharing using digital aids to ensure Māori can readily access BD healthcare from any service/team/region if acutely unwell.
• Use digital technology to track service adherence to established best practice treatment guidelines for BD to monitor health equity for Māori patients and whānau.

#### 3.5.3 Quality improvement‐focussed

Participants reported experiencing negative outcomes perpetuated by poor information management and outdated patient health information systems, leading to a lack of proactive information sharing or supportive follow up.

I suffered post‐natal depression but it was never picked up. I was admitted, and then they just let me out. Nobody followed me up. I was back out with nothing, young mum, homeless with absolutely no support. I wasn't on medication. I needed help. A lot of my trouble started with that. (P15)

Negative experiences raised by Māori with BD and their whānau highlighted the potential for information management and technology to be better utilised to collect quality health information and learn from systemic failures to improve the organisation of healthcare.

I went to the doctors seven times in a fortnight and said there is something wrong with me and she said – no, everything is fine. I should have gone into respite care so I could come down off the mania, but instead I just stayed high for about six months. (P5)

Participants also identified that the MHS needed to use information management and technology to improve the quality of BD care, by actively monitoring symptoms to guide treatment and proactively prevent the risk of relapse. The lack of effective use of information management and technology meant that Māori patients and whānau experienced delays in receiving quality health care because clinicians or services relied on their limited knowledge or recall of the case history. Better use of information management and technology could improve the timeliness and tailoring of BD interventions by identifying patterns in the onset of symptoms, relapse of illness, and efficacy of interventions provided to Māori patients and their whānau.

There was no follow‐up by the GP to see how he was actually going with those meds. Then he had a full‐blown psychotic episode. We saw the mental health team and I explained everything but they didn't know what he was normally like, and didn't account for other symptoms of psychosis. They said he was fine, because he was manic, not suicidal. (W4)

Participants' critique of information management and technology revealed opportunities for change to improve the quality of the organisation of healthcare. Attributes of an organisation redesigned to use information management and technology to track healthcare quality improvements supporting equitable healthcare for Māori with BD and their whānau are presented in Table 16.

**TABLE 16:** Information management and technology to improve the quality of healthcare

• Monitor the care of Māori patients with BD in the H&DS to alert clinicians to relevant health information, ensuring patients are not lost to treatment/follow‐up.
• Track declined referrals by services to proactively inform organisational healthcare quality improvements, enhance healthcare access, and prevent harm to Māori patients with BD and their whānau.
• Monitor adverse outcomes of service contact and record triggers to relapse for Māori patients with BD to inform healthcare quality improvements to organisational culture/design/incentive structures.

## 4 DISCUSSION

This study utilised an adapted analytic frame to explore barriers to equity and propose changes to the organisation of healthcare in New Zealand for Māori with BD and their whānau.^5^, ^10^ Participants highlighted the need for transformation across the organisation of healthcare to achieve health equity objectives for Māori. Executive Management changes need to establish an equity partnership model, embed cultural safety and redesign healthcare to enhance wellbeing for Māori. Incentive structures need to diversify, develop and retain a culturally competent health workforce, and utilise information management and technology to guide whole system improvements.

These findings are consistent with research that found policy changes would not lead to health equity in New Zealand unless healthcare organisations were properly funded and resourced, and equity gains for Māori were measured, incentivised and enforced.^1^ Findings also aligned with international critique of planned healthcare reforms, noting that quality improvement efforts would not produce equity without addressing organisational factors that perpetuate unequal access to and through quality healthcare for minoritised peoples.^7^ This paper expands on existing health equity frameworks describing how leadership and quality outcome measures could be used to guide changes to the organisational culture, design and incentive structures of healthcare to achieve equity for Māori with BD.^12^, ^22^

Our findings are also consistent with broadly stated objectives of planned health reforms in New Zealand that are yet to be comprehensively detailed.^23^ Planned transformations include: express commitments to Treaty partnerships between Māori and the Crown; establishing a Māori Health Authority to lead reforms of an equitable system; and goals to deliver greater access, experience and outcomes of care for Māori.^23^ Incorporating findings from this paper provides an opportunity to apply the expertise of Māori patients and whānau to planned reforms of the organisation of healthcare and prevent the perpetuation of current inequities in New Zealand. Given similar health inequities are experienced by Indigenous peoples relative to benchmark populations worldwide, this paper may have international implications.^8^, ^24^

Strengths of this study include the KMR design in a previously under researched area, and adaptation of a method that privileges the expertise of Māori identifying barriers and potential solutions to improve the organisation of healthcare for BD in New Zealand. Recruiting Māori patients and whānau through services may have limited participation to people with positive experiences, however, this did not appear to be reflected in interview data. In addition, if time had allowed, separate interviews with patients and whānau may have highlighted different critique between groups, however, the benefits of one interview were considered and aligned with KMR principles.^13^

## 5 CONCLUSION

The organisation of healthcare requires transformation to achieve health equity outcomes in New Zealand.^9^, ^12^ Clinical and structural changes are also required to address systemic barriers that prevent health equity for Māori, this paper highlights the full extent of organisational change required across the H&DS.^1^, ^25^, ^26^ The organisation of healthcare, while known to effect the processes and outcomes of care, has been under‐researched, and under‐addressed privileging the needs of majority populations by design, leadership, and models of delivery.^4^, ^5^, ^6^ The challenge going forward is whether the resourcing for an equitable healthcare organisation will be implemented across the H&DS in partial fulfilment of the long overdue promises of the Crown.

## CONFLICT OF INTEREST

The authors report that there are no competing interests to declare.

## Supporting information

Supplementary Table S1

## Data Availability

Research data are not shared.
