# Supplementary material for: “If we can just dream…” Māori talk about healthcare for bipolar disorder in New Zealand: A qualitative study privileging Indigenous voices on organisational transformation for health equity
Source: Int J Health Plann Manage. 2022 Apr 23;37(5):2613–34. doi: 10.1002/hpm.3486 (PMC9546144; doi:10.1002/hpm.3486)
Supplement: Supplementary file 1 — Supplementary Table S1 [file HPM-37-2613-s001.docx]

**Supplementary Table 1: Integrated Coding Framework**

| **Organisational Health Quality Improvement Principles ^5^** | **Whakamaua: Māori Health Action Plan (2020-2025) ^10^** | **Coding Definition** |
| --- | --- | --- |
| **Executive Management (EM):** Senior leadership and board take responsibility to articulate and communicate mission statement, goals, and objectives. These are directed, led, and supported by the Executive Management team. A commitment to health quality improvement (HQI) is evidenced by behaviour of the EM team, and allocation of roles at all levels of the health system. Involves monitoring of HQI and overt accountability for outcomes with a budgetary investment in monitoring/reporting and policy commitment to HQI. | EM leadership team and board are committed to health equity for Māori. EM team includes Māori leaders and contributes to the development of Māori to lead across all levels of the health and disability system (H&DS) and guide HQI/equity for Māori. Mātaurangā Māori knowledge is recognised and valued by EM team as essential for Māori health equity. Māori health data and Kaupapa Māori research (KMR) findings are closely monitored to inform HQI/equity outcomes. KMR findings are embedded by EM into policy, OC, OD and IS to integrate mātaurangā Māori in H&DS. Budgetary commitment to equity for Māori allows adjustments to funding models to address unmet needs. | EM include Māori senior leaders and support Māori leader development. Equity for Māori in H&DS prioritised, communicated and reflected in dedicated equity-focused roles across health. EM understand mātauranga Māori and Westernised approaches and integrate both in H&DS policy. Commitment to equity funding model/KMR to refine HQI/equity gains. |
| **Organisational Culture (OC)**: Values, assumptions and beliefs of the health system that inform definition of HQI. Investment in quality management training of EM and team leaders. Commitment to accountability embedded throughout the system but modelled and normalised by the EM team. Evaluation, implementation, monitoring, and revising are built into the organisational culture to create lasting HQI. Commitment to cross-service and within-teams communication embedded into OC to maximise limited resources. | The OC commits to principles of the Treaty of Waitangi (TToW) and recognises that H&DS contributes to health inequities. The OC acknowledges there are unfair, avoidable deficits in Māori health and seeks to address these through organisational change to a culture that understands, values, and utilises mātauranga Māori and tikanga Māori for HQI/equity of outcome. Cross-sector/service /agency and ‘whole of government’ work is established and prioritised for HQI/equity to address social and Indigenous specific determinants of health. Requirement for services to publish Māori HQI/equity plans & progress to demonstrate the commitment to change. | Organisation adopts a culture of change to reduce health inequities unfairly impacting Māori/ contribution of H&DS to these. Teams have ongoing training/resources to integrate mātauranga, tikanga and Māori models of health in/across the system and reviews/publishes/ improves equity gains and normalises accountability. |
| **Organisational Design (OD)**: Branch within the health system dedicated to and responsible for HQI. HQI strategists are linked in with all parts of the system not limited by centralisation. HQI is defined by staff with specialist skills to ensure HQIs are implemented at clinical levels and not just reflected in policy. OD is flexible to ensure HQI evolves from top-down, bottom-up iterative process. HQI leaders are positioned in all departments and service lines (i.e. clinical, administrative, etc). | H&DS are designed to recognise the interdependent relationships between people, whānau, and social contexts and their impact on HQI/equity for Māori. HQI/equity improvement for Māori is a shared responsibility and will be monitored by the Ministry of Health (MOH) who will adapt the OD based on identified needs through engaging with sovereign Māori partners. There will be greater Investment in Iwi and the Māori health sector to deliver local, holistic, integrated whānau-centred KM H&DS. The OD includes cross-service partnerships that position Māori patients/whānau in the centre of co-ordinated/ integrated culturally safe/responsive care. | Equity roles/goals/gains are visible in KM & H&DS teams. Equity measures are used in partnership with Māori to refine OD/achieve equity. Cultural competence/safety training needs are met, and equity gains/outcomes are measured and shared. OD includes whānau-centred/ cross-service partnerships to provide holistic care. |
| **Incentive Structures (IS)**: Design of HQI oriented Human Resource (HR) processes that measure performance of managers, services, teams and clinicians against established quality standards and outcomes. Utilises a balanced scorecard, to move the focus from cost – to cost-benefit analysis by measuring progress in financial, customer, process, learning and growth scores. IS align salary and career progression with key performance indicators measured by HQI outcomes. | The MOH will monitor health outcomes for Māori and measure the quality of partnerships between Māori and the Crown to drive improvements. Iwi and hapū will be resourced to develop KM Services by, for, and with their communities. The H&DS will be resourced to attract, develop and retain a skilled Māori health workforce. Mātauranga Māori will be recognised and valued as a professional skill of relevance to HQI/equity. Professional bodies and H&DS will invest in anti-racism/anti-discrimination/cultural competence/ cultural safety skills as essential to HQI/equity. H&DS will be routinely monitored against HQI/equity standards aligned with Māori perspectives of health. | HR performance plans/ appraisals include measures of quality of partnerships with Māori; knowledge and application of mātauranga, tikanga and Māori models of health; uptake of equity training/skills/standards of care; contribution towards team and wider H&DS equity improvements. Professional bodies prioritise equity for Māori in registration standards. |
| **Information Management & Technology (IMT)**: Investment in technology to monitor, manage, and share HQI information in a timely way. National investment in HQI data management system to reduce cost to individual services. National HQI data collection service that is reliable, consistent, and comparable. Real time feedback to maximise HQI learning. | The MoH will ensure HQI/equity for Māori by: increasing evidence supporting the efficacy of rongoā/mātauranga Māori; investing in national communication campaigns; screening and immunisation programmes; and service monitoring tools tailored to measure HQI/equity for Māori. Prioritising collection of quality KM health data analysed using a Māori data sovereignty approach for HQI/equity. Innovative technology will be adopted nationally to monitor HQI/equity/continuity of care to allow a whole-of-government/cross-service view of equity gains. | National data quality/ integrity/tech/measurement prioritised to support timely collection of quality ethnicity and health data, rapid analysis, and communication about equity gains across and within H&DS. Data collection, analysis and communication led by Indigenous data sovereignty/KMR experts. |
